# Investigating the Role of *B9D1* in Meckel–Gruber Syndrome: A Case Report and Comprehensive Literature Review

**DOI:** 10.3390/genes16060643

**Published:** 2025-05-27

**Authors:** Gianluca Campobasso, Ludovica Mercuri, Francesca De Razza, Antonella Cosentino, Marta Mele, Antonella Monittola, Carmen Congedo, Maria Chiara Calò, Caterina Scalcione, Alessandro D’Amuri, Salvatore Mauro, Serena Lattante

**Affiliations:** 1 Fetal Medicine Service, Department of Obstetrics and Gynecology, Vito Fazzi Hospital, 73100 Lecce, Italy; gianlucacampobasso1@gmail.com; 2 Department of Experimental Medicine, University of Salento, 73100 Lecce, Italy; ludovica.mercuri@unisalento.it (L.M.);; 3 Medical Genetics Unit, Presidio Ospedaliero “Vito Fazzi”, 73100 Lecce, Italygenetica.polecce@asl.lecce.it (S.M.); 4 Department of Obstetrics and Gynecology, University of Bari, 70121 Bari, Italy; 5 Anatomic Pathology Unit, Presidio Ospedaliero “Vito Fazzi”, 73100 Lecce, Italy

**Keywords:** MKS, Meckel–Gruber syndrome, *B9D1*, encephalocele, ciliopathy

## Abstract

Meckel–Gruber syndrome (MKS) is a rare autosomal recessive lethal ciliopathy, characterized by occipital encephalocele, cystic kidneys, and postaxial polydactyly, caused by mutations in different genes. Its significant genetic heterogeneity along with its clinical overlap with other ciliopathies makes early diagnosis essential for clinical management, accurate genetic counseling, and informing future reproductive decisions. Objectives: This study aims to describe a prenatally diagnosed case carrying a homozygous *B9D1* variant and to examine the current literature on all variants reported in this gene associated with MKS. Methods: We comprehensively review the current literature on pathogenic *B9D1* variants implicated in this syndrome. Additionally, we describe a case, presenting multiple congenital anomalies suggestive of MKS, genetically diagnosed by clinical exome sequencing on chorionic villi. Results: Occipital encephalocele and polycystic kidneys were revealed via ultrasound, thus suggesting MKS. Genetic testing identified the homozygous c.151T>C (p.Ser51Pro) variant in the *B9D1* gene, inherited from healthy parents. Conclusions: This case supports the pathogenicity of the homozygous *B9D1* c.151T>C variant and underscores the importance of timely prenatal assessment and targeted genetic testing for the detection of MKS risk in heterozygous subjects, enabling appropriate pregnancy management and informed reproductive choices.

## 1. Introduction

Meckel–Gruber syndrome (MKS, also known as MGS, OMIM: 614209) is a rare, lethal, autosomal recessive disorder. It is primarily characterized by a triad of congenital malformations: occipital encephalocele, cystic dysplasia of the kidneys, and postaxial polydactyly. These alterations are usually detected by prenatal ultrasound mostly in the late first trimester [1]. Central Nervous System abnormalities are considered a consistent hallmark of MKS, though their manifestation can widely vary. Additional features, observed in approximately 30% of fetuses, may include anencephaly or microcephaly, the molar tooth sign (MTS), craniofacial malformations, a cleft lip and palate, short and curved long bones, congenital heart defects, pulmonary hypoplasia, and male genital defects [2,3,4,5]. Rare features, occurring in fewer than 20% of cases, include cystic dysplasia of the lungs and thyroid, retinal colobomas, and situs defects [3,5,6,7,8].

The worldwide incidence of MKS varies from 1 in 13.250 to 1 in 140.000 live births [9] with no differences between males and females [10]. The highest incidence, estimated at 1 in 1.300 live births with a carrier frequency of approximately 1 in 18, was reported among Gujarati Indians in a perinatal mortality survey conducted between 1976 and 1982 [11]. Consistently with these data, a particularly high gene frequency for MKS was subsequently reported in the Asian Hindu population of Leicestershire, originally derived from the Gujarat region in western India [12].

Meckel–Gruber syndrome is classified as a ciliopathy, arising from defects in primary cilia, which are critical for cell signaling during development. Specifically, it is considered the most severe condition in this spectrum, typically leading to death in utero or shortly after birth. The primary cilium is a microtubule-based projection extending from the apical surface of vertebrate cells. It serves as a sensory hub, detecting and relaying chemical and mechanical signals while also orchestrating key signaling pathways—such as Wnt (Wingless-Type MMTV integration site family) and Shh (Sonic hedgehog)—that are essential for embryogenesis, intracellular signaling, and proper organ formation. Many of the proteins associated with MKS are localized to the transition zone (TZ), a specialized region of the cilium that controls the selective trafficking of molecular components, including proteins and lipids [13].

Ciliopathies are characterized by extreme genetic heterogeneity, involving genes that encode proteins crucial to the primary cilium’s architecture and function, which complicates diagnosis. Notably, the recent advancement in routine-use multi-gene panels in molecular testing has strongly improved both genetic diagnosis and the clinical management of families affected by the disease.

The recognition of MKS is crucial not only for enabling an early and accurate prenatal diagnosis, followed by appropriate management of an affected pregnancy, but also for providing accurate genetic counseling and informing future reproductive decisions. In this paper, we present a review of the current state of scientific knowledge on pathogenic and likely pathogenic variants in the *B9D1* gene, which are known to be causative of Meckel–Gruber syndrome. Furthermore, we describe a prenatally diagnosed case of MKS, which, moreover, led to the discovery of the parents’ status as disease-causing mutation carriers. Genetic diagnosis played a key role in shaping their understanding of the condition and in guiding their choices for future pregnancies.

## 2. Case Report

### 2.1. Clinical Case

**Figure 1:**
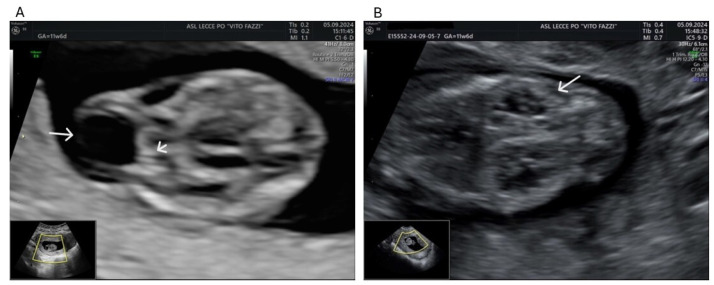
(**A**) The picture shows the occipital cranial bone defect (white arrowhead) with herniated meninges (white arrow). (**B**) Markedly enlarged and heterogeneous kidneys are shown. An unusual corticomedullary differentiation is evident with the appearance of small pyramidal cysts. Multiple diffuse cystic lesions, surrounded by a more homogeneous echogenic rim corresponding to the cortex (white arrow), are evident.

A 30-year-old woman was referred to our center for suspicion of brain anomaly and kidney malformations in her fourth pregnancy. According to ISUOG Practice Guidelines (2023), an ultrasound performed at 11 + 6 weeks of gestation showed a vital fetus with a crown rump length of 46.4 mm. Nuchal translucency was 1.4 mm, and nasal bone was present. Axial and sagittal planes showed an occipital defect with the protrusion of meninges. An occipital cranial bone defect with a herniated brain-filled cyst was detected (Figure 1A) as well as the absence of one of the three posterior brain spaces. Kidneys appeared enlarged with a polycystic aspect and unusual corticomedullary differentiation (Figure 1B). Chorionic villus sampling was performed at 13 weeks, and the voluntary termination of pregnancy (VTP) was performed, regardless of genetic results.

**Figure 2:**
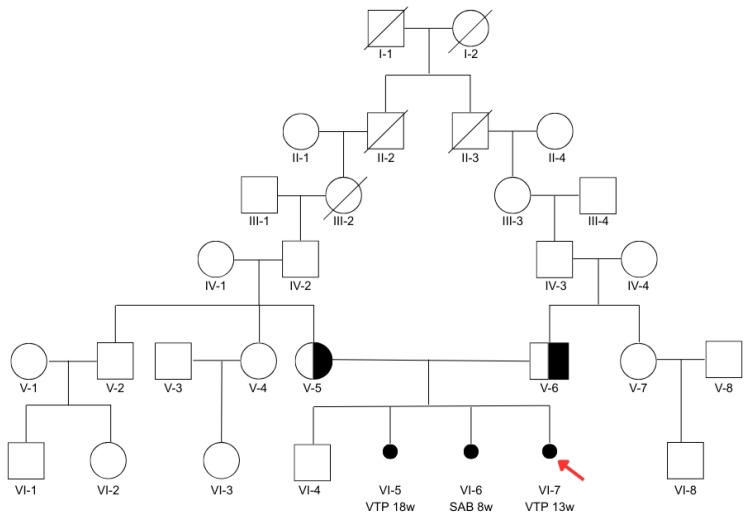
Pedigree of the family. The red arrow indicates the index case (VI-7). VTP: voluntary termination of pregnancy; SAB: spontaneous abortion; w: gestational age in weeks.

The parents (V-5 and V-6, Figure 2) report having a firstborn healthy male child (VI-4, Figure 2), currently 5 years old, born at term by cesarean section, followed by a termination of pregnancy for fetal anomalies in the second trimester (VI-5, Figure 2) and a spontaneous abortion at 9 weeks of gestation (VI-6, Figure 2). Specifically, the second pregnancy was interrupted at 18 weeks, and the fetus was examined, with observations including bilateral postaxial polydactyly with six toes on both feet and six fingers on the left hand, ventriculomegaly, and polycystic kidneys. Both parents are phenotypically healthy and have no clinical features detectable through routine medical analyses. They deny any family history suggestive of Joubert syndrome or Meckel–Gruber syndrome as well as any case of spontaneous abortion (SAB) or volunteer terminations of pregnancy due to prenatal anomalies. A deep investigation of the family history revealed that the partners, both originating from the same village, share a background of consanguinity: their paternal grandmothers (III-2 and III-3, Figure 2) were first cousins through their paternal lineage.

### 2.2. Genetic Analysis

#### 2.2.1. Methods

Chorionic villus sampling was performed at 13 weeks for fetal karyotyping by QFQ banding (400-band resolution). After written informed consent was obtained, fetal DNA was extracted from chorionic villi while parental DNA was extracted from blood, using the Libex Nucleic Acid Extractor (Tianlong, China). Clinical exome sequencing, targeting 4800 disease-associated genes, was performed. Libraries were prepared using TruSight One Sequencing Panels (Illumina, San Diego, CA, USA) and sequenced using the NextSeq 500/550 v2.5 kit (Illumina) on the NextSeq 500 Sequencing System (Illumina). Raw data were analyzed using VarSome Clinical v.12.6.1. An average coverage of at least 40× and allelic balance > 0.24 were obtained for each sample. Data were filtered to analyze the following genes, associated with MKS: *AHI1*, *B9D1*, *B9D2*, *CC2D2A*, *CEP290*, *MKS1*, *NPHP3*, *RPGRIP1L*, *TCTN1*, *TCTN2*, *TMEM138*, *TMEM216*, *TMEM237*, *TMEM67*, *TTC21B*, and *WDPCP*. Only exonic variants and variants involving exon–intron junctions with MAF < 0.10, according to gnomAD v4.1 (https://gnomad.broadinstitute.org accessed on 27 April 2025), were considered.

#### 2.2.2. Genetic Findings

A normal female karyotype was detected on chorionic villi. The clinical exome revealed the homozygous missense variant c.151T>C in the *B9D1* gene, consisting of the replacement of the Serine at position 51 with a Proline (p.Ser51Pro). A genetic analysis of the parents revealed that both were carriers of the variant in a heterozygous state. Therefore, the missense mutation, inherited by the fetus from healthy heterozygous parents, is extremely rare in the control population, with an allelic frequency of 0.00005778 (gnomAD, https://gnomad.broadinstitute.org). It involves a conserved residue (phyloP100: 5.384) and is considered likely pathogenic by in silico predictors, according to ACMG guidelines (PM3, PM2, PP3).

## 3. Exploration of the Literature on *B9D1* in MKS

The *B9D1* gene, mapped to chromosome 17p11.2 [14], was first known as the *MKS1* gene. It contains a typical and intriguing B9 domain, exclusively found in a family of three proteins associated with basal bodies and primary cilia in mammals: MKS1, MKS1-Related-Protein-1 (MKSR1), and MKS1-Related-Protein-2 (MKSR2). The B9 domain encompasses amino acids 11 to 174 and is essential for ciliogenesis and ciliary protein localization [15].

The *B9D1* gene was first associated with MKS in 2011, after the conducting of Next-Generation Sequencing (NGS) targeting 31 ciliopathy-associated genes in 12 MKS-affected families. Among these pedigrees, one included a fetus diagnosed at 13 weeks of gestation via ultrasound with multiple anomalies: posterior encephalocele, an abnormal posterior fossa, bilaterally enlarged multicystic dysplastic kidneys, and an absent bladder. Although polydactyly, a hallmark of MKS, was not observed, the fetus presented with bilateral clubfoot and shortened limbs. The pregnancy continued until 35 weeks of gestation; however, the neonate survived only a few hours after birth. Trio-based NGS revealed a paternally inherited splicing mutation in *B9D1* (c.505+2T>C). Since Sanger sequencing suggested apparent hemizygosity of the variant in the fetus, array-CGH analysis was performed, thus identifying a de novo 1.713 Mb deletion encompassing the *B9D1* locus. Additionally, the fetus carried a maternally inherited variant in another MKS gene, *CEP290* (c. 6628C>T; p.R2210C) [16]. The identification of a likely pathogenic variant in the *CEP290* gene, alongside the biallelic in *trans* alteration in *B9D1*, raised suspicions regarding a possible oligogenic contribution to the severity of the observed phenotype. Mutations in *CEP290*, encoding a centrosomal protein essential for ciliary assembly and trafficking, have been associated with several ciliopathies, including Bardet–Biedl syndrome 14 (OMIM 615991), Joubert syndrome (OMIM 610188), and Meckel syndrome type 4 (OMIM 611134). The importance of *CEP290* as a causal gene in MKS is well documented [17,18]. Therefore, the presence of the likely pathogenic *CEP290* variant in this genetic context may represent a modifying or contributing allele to the phenotype severity, aligning with the previously described concept of oligogenic inheritance in ciliopathies [19,20,21]. Accordingly, a patient with Joubert syndrome (JBTS), carrying a heterozygous mutation in the *CC2D2A* gene and two variants in the *B9D1* gene, was described by Kroes and colleagues [22]. The c.949G>A; p.Gly317Arg variant in the *CC2D2A* gene (NM_001080522.2) was predicted to be probably damaging and deleterious by the in silico tools PolyPhen and SIFT. Additionally, the *B9D1* variants were the missense c.151C>T; p.Ser51Pro, predicted to be deleterious, and the non-coding variant c.473-52G>T, predicted to not affect splicing. Based on an alternative transcript of the gene (ENST00000395616), the variant would result in an amino acid substitution (p.Trp170Cys), although predicted to be benign. According to current ACMG guidelines, the variant is classified as of uncertain significance (PM2, BP7). Parental DNA was not available to determine the haplotype phase of the two *B9D1* variants. However, the identification of patients carrying heterozygous likely pathogenic variants in *CEP290* or *CC2D2A* combined with deleterious mutations in *B9D1* may support a digenic or triallelic model of inheritance, particularly given the known functional interaction between the encoded proteins. Indeed, *CEP290*, *CC2D2A*, and *B9D1* are components of the B9 multiprotein complex localized at the basal body, specifically within the transition zone at the base of the cilium. This complex acts as a selective diffusion barrier, regulating the trafficking of proteins between the plasma membrane and the ciliary membrane. These findings suggest that variants in different ciliary genes may modulate clinical expressivity, thereby broadening the genetic and pathogenic landscape of the syndrome. In 2014, two additional patients were reported with the clinical diagnosis of JBTS. The first case was a 9-year-old boy presenting with hypotonia, ataxia, developmental delay, intellectual disability, oculomotor abnormalities (OMAs), nystagmus, MTS, and dysmorphisms, with the homozygous c.467G>A; p.(Arg156Gln) variant in *B9D1*. The second patient, a 7-year-old girl, showed a phenotype closely resembling that of the male patient, with no intellectual impairment, and compound heterozygosity for two inherited *B9D1* variants: c.95A>G; p.(Tyr32Cys) and c.520_522delGTG; p.(Val174del) [23].

In 2015, a genetic analysis performed of a 22-year-old patient with MTS, OMA, hypotonia, ataxia, congenital clubfoot, and dysphasia identified two variants in *B9D1* predicted to be damaging: a maternally inherited nonsense mutation (c.493C>T; p.Gln165*) and a de novo missense mutation (c.151T>C; p.Ser51Pro) [24].

Two additional patients with mutations in *B9D1* were identified, one homozygous for the variant c.285C>A; p.(Phe95Leu) and another one carrying the previously reported c.95A>G variant in compound heterozygosity with a novel change, c.466C>T; p.(Arg156Trp) [25]. Interestingly, this same combination of variants was subsequently reported in a 5-year-old patient, evaluated at the NIH Clinical Center, presenting with MTS, vermis hypoplasia, OMA, nystagmus, and an absence of speech [26,27].

An insightful correlation between antenatal ultrasound findings, postnatal renal imaging, and targeted exon sequencing using a renal gene panel was described in 2016. The study reported a fetus presenting with encephalocele, renal cysts, and polydactyly, where NGS identified a homozygous inherited variant in *B9D1* (c.508_510delCTC), resulting in the in-frame deletion of a leucine residue at position 170, being lethal to the fetus [28].

In 2020, a 24-year-old woman with a JBTS phenotype was described, with MTS, global developmental delay, delayed ambulation, poor balance, head tilt, rotational nystagmus, dysarthria, and hypometric saccades. She carried biallelic inherited variants in *B9D1*: c.341G>A; p.(Arg114Gln) and c.529G>C; p.(Asp177His), detected by clinical exome sequencing [29].

To our knowledge, this is the first time that the missense variant c.151T>C has been detected in a homozygous state, yielding new information about the genotypic spectrum of *B9D1*-related ciliopathies. All presented cases are summarized in Table 1, with regard to their genetic findings, and in Table 2, with respect to their clinical phenotypes.

## 4. Discussion

Our comprehensive review of the literature highlights the emerging role of *B9D1* in the pathogenesis of ciliopathies in the clinical and scientific landscape over the past fifteen years. Thus, we included only pathogenic and likely pathogenic *B9D1* variants, integrating data from the published scientific literature as well as from the Leiden Open-source Variation Database v.3.0 (LOVD; https://www.lovd.nl/, accessed on 27 April 2025), the Human Gene Mutation Database (HGMD^®^; https://www.hgmd.cf.ac.uk/ac/index.php, accessed on 27 April 2025), and ClinVar (https://www.ncbi.nlm.nih.gov/clinvar/) (accession date: 27 April 2025). A total of 12 variants have been reported to be associated with a phenotypic spectrum extending from the lethal MKS to the non-lethal JBTS. The clinical overlap between these two conditions, while posing significant challenges for differential diagnosis, also offers valuable insights into the pathophysiology of ciliary proteins and their interactions in determining disease onset and severity.

**Figure 3:**
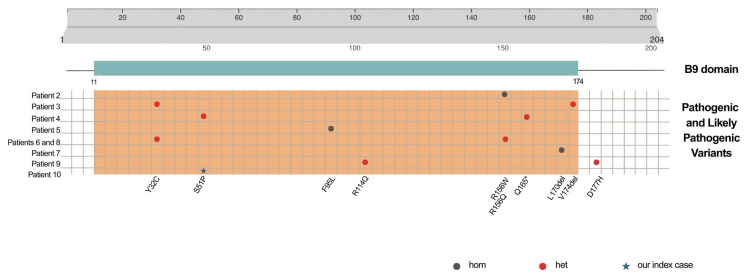
B9d1 domain is shown. Gray bars represent the full-length 204-amino-acid protein encoded by the canonical transcript NM_015681.6/ENST00000261499.11. The green bar indicates the B9 domain, the sole domain of the protein, spanning amino acids 11 to 174, as annotated by InterPro (https://www.ebi.ac.uk/interpro/, accessed on 27 April 2025). In the orange panel, pathogenic and likely pathogenic variants—identified in patients with MKS/JBTS phenotypes reported to date in the literature, including our case—are shown. A red dot indicates a heterozygous variant, a dark green dot denotes a homozygous variant, and the star marks our index case.

Furthermore, all described cases, including the one we report here, carry *B9D1* variants lying in the unique B9 domain, essential for ciliogenesis, thus emphasizing its important role and the substantial phenotypic impact of these mutations. Exclusively, one patient (number 9, Table 1) has been reported with the compound heterozygosity of two different variants, p.P114Q and p. D177H; notably, while the former falls in the B9 domain, the latter is located three amino acids downstream (Figure 3).

Here we report a case that underscores the critical importance of a synergistic approach, combining early clinical diagnosis with targeted molecular analysis, to uncover the genetic etiology underlying MKS and to assess recurrence risk within the family. Our case provides additional evidence supporting the pathogenicity of the *B9D1* c.151T>C (p.Ser51Pro) variant, for the first time in homozygosity, in the context of MKS. This variant, previously described exclusively in compound heterozygosity in a patient with JBTS, now expands the known genotypic and phenotypic spectrum of *B9D1*-related ciliopathies. The identification of a genetic variant in a couple with a history of recurrent pregnancy malformations further emphasizes the essential and unique role of molecular diagnosis. This approach not only helps to clarify the genetic cause of congenital anomalies in the ongoing pregnancy but also provides valuable insights for future reproductive planning. A precise genetic diagnosis enables clinicians to better assess the recurrence risk in future pregnancies and to offer targeted strategies, such as the possibility of Preimplantation Genetic Testing (PGT) or early prenatal diagnosis. These opportunities should always be discussed with the couple in the context of appropriate genetic counseling to permit the most appropriate and informed decision-making.

Finally, accurate clinical assessment and timely genetic investigation are essential for identifying *B9D1* variants causative of MKS. These steps are crucial for improving our understanding of the *B9D1*-associated phenotypic continuum and for supporting clinicians and researchers in refining clinical and molecular knowledge of this rare inherited disorder.

## Figures and Tables

**Table 1:** Curated overview of pathogenic and likely pathogenic *B9D1* variants associated with Meckel–Gruber syndrome from the literature, integrated with our case.

Patient Number	DNA Change	Protein Change	Transcript	Segregation	Zygosity	Other Variants	Ref.
1	c.505+2T>C	–	NM_015681	pat	hetx	CEP290 c.6628C>T	[16]
	17p11.2del	–		de novo	het		
2	c.467G>A	R156Q	NM_015681	inher*	hom	–	[17]
3	c.95A>G	Y32C	NM_015681	inher*	het	–	[17]
	c.520_522del	V174del		inher*	het		
4	c.493C>T	Q165*	**NM_001321218**	mat	het	–	[18]
	c.151T>C	S51P		de novo	het		
5	c.285C>A	F95L	NM_015681	NA	hom	–	[19]
6	c.95A>G	Y32C	NM_015681	NA	het	–	[19]
	c.466C>T	R156W		NA	het		
7	c.508_510del	L170del	NM_015681	pat/mat	hom	–	[22]
8	c.95A>G	Y32C	NM_015681	**mat**	het	–	[20,21]
	c.466C>T	R156W		**pat**	het		
9	c.341G>A	R114GQ	NM_015681	inher*	het	–	[23]
	c.529G>C	D177H		inher*	het		
10	c.151T>C	S51P	NM_015681	pat/mat	hom	–	Present paper

Inher*: not specified whether maternally or paternally inherited; pat: paternal inheritance; mat: maternal inheritance; het: heterozygous; hom: homozygous. In bold: curated information added in this paper.

**Table 2:** Clinical findings of patients described in Table 1.

Patient Number		1	2	3	4	5	6	7	8	9	10
Gender		M	M	F	M	NA	NA	NA	M	F	M
Prenatal Phenotype	Encephalocele	✔						✔			✔
	Multicystic kidneys	✔						✔			✔
	Polydactyly							✔			
	MTS		✔	✔	✔				✔	✔	
	OMA		✔	✔	✔				✔		
	JBTS phenotype		✔	✔		✔	✔			✔	
	Vermis hypoplasia								✔		
	Clubfeet	✔			✔						
Postnatal Phenotype	Age at observation	1.75 h	9 yo	7 yo	22 yo	NA	NA	-	4.8 yo	24 yo	-
	Hypotonia		✔	✔	✔						
	Nystagmus		✔	✔					✔	✔	
	Ataxia		✔	✔	✔						
	Developmental delay		✔	✔						✔	
	Dysmorphisms		✔	✔							
	Ambiguous genitalia	✔									
	Dysphasia				✔						
	Absent speech								✔		
	Hypometric saccades									✔	
	Dysarthria									✔	
	Head tilt									✔	

M: male; F: female; NA: information not available; “-”: lethal to fetus; h: hours; yo: years old; MTS: molar tooth sign; OMA: oculomotor apraxia; JBTS: Joubert syndrome.

## Data Availability

No new data were created.

## Abbreviations

The following abbreviations are used in this manuscript:

MGS/MKS	Meckel–Gruber Syndrome
JBTS	Joubert Syndrome
MTS	Molar Tooth Sign
OMA	Oculomotor Apraxia
Inher	Inherited
Pat	Paternal
Mat	Maternal
Hom	Homozygous
Het	Heterozygous
TZ	Transition Zone
PGT	Preimplantation Genetic Testing
NGS	Next-Generation Sequencing
VTP	Voluntary Termination of Pregnancy
SAB	Spontaneous Abortion
W	Gestational age in weeks
M	Male
F	Female
h	Hours
yo	Years Old
NA	Not Available
OMA	Oculomotor Apraxia
